# Leveraging Neuroimaging Tools to Assess Precision and Accuracy in an Alzheimer’s Disease Neuropathologic Sampling Protocol

**DOI:** 10.3389/fnins.2021.693242

**Published:** 2021-08-18

**Authors:** Jason M. Webster, Thomas J. Grabowski, Tara M. Madhyastha, Laura E. Gibbons, C. Dirk Keene, Caitlin S. Latimer

**Affiliations:** ^1^Department of Radiology, University of Washington, Seattle, WA, United States; ^2^Department of Neurology, University of Washington, Seattle, WA, United States; ^3^Department of Medicine, University of Washington, Seattle, WA, United States; ^4^Department of Laboratory Medicine and Pathology, University of Washington, Seattle, WA, United States

**Keywords:** Alzheimer’s disease, neuropathology, neuroimaging, functional connectivity, accuracy, precision

## Abstract

**Introduction:**

The study of Alzheimer’s disease investigates topographic patterns of degeneration in the context of connected networks comprised of functionally distinct domains using increasingly sophisticated molecular techniques. Therefore, obtaining high precision and accuracy of neuropathologic tissue sampling will enhance the reliability of molecular studies and contribute to the understanding of Alzheimer’s disease pathology. Neuroimaging tools can help assess these aspects of current sampling protocols as well as contribute directly to their improvement.

**Methods:**

Using a virtual sampling method on magnetic resonance images (MRIs) from 35 participants (21 women), we compared the precision and accuracy of traditional neuropathologic vs. neuroimaging-guided sampling. The impact of the resulting differences was assessed by evaluating the functional connectivity pattern of regions selected by each approach.

**Results:**

Virtual sampling using the traditional neuropathologic approach had low neuroanatomical precision and accuracy for all cortical regions tested. Neuroimaging-guided strategies narrowed these gaps. Discrepancies in the location of traditional and neuroimaging-guided samples corresponded to differences in fMRI measures of functional connectivity.

**Discussion:**

Integrating neuroimaging tools with the neuropathologic assessment will improve neuropathologic-neuroimaging correlations by helping to ensure specific functional domains are accurately sampled for quantitative molecular neuropathologic applications. Our neuroimaging-based simulation of current sampling practices provides a benchmark of precision and accuracy against which to measure improvements when using novel tissue sampling approaches. Our results suggest that relying on gross landmarks alone to select samples at autopsy leads to significant variability, even when sampled by the same neuropathologist. Further, this exercise highlights how sampling precision could be enhanced if neuroimaging were integrated with the standard neuropathologic assessment. More accurate targeting and improved biological homogeneity of sampled brain tissue will facilitate the interpretation of neuropathological analyses in AD and the downstream research applications of brain tissue from biorepositories.

## Introduction

Much of our current understanding of Alzheimer’s disease (AD) pathophysiology is derived from the neuropathologic comparison of brains from individuals diagnosed with AD dementia in life with brains from cognitively normal individuals. Neuropathologic evaluation allows for a deep characterization of the pathology at the tissue, cellular, and molecular levels, but is typically limited, for pragmatic reasons, to a few small samples of each brain. Current, widely practiced guidelines for the neuropathologic assessment of AD recommend sampling four regions of cerebral cortex [middle frontal gyrus (MFG), inferior parietal lobule (IPL), superior/middle temporal gyri, and calcarine cortex] from coronal brain slices for histopathologic, biochemical, and molecular assessments (Montine et al., 2012). Together these samples constitute a miniscule fraction of the total brain volume. Gross anatomical landmarks guide sampling of these regions. For example, the MFG and superior/middle temporal gyri are sampled on the coronal slice containing the anterior commissure. This approach provides some degree of consistency, but the degree to which the sampled regions correspond to the same structural and functional brain area across individuals is unknown.

Providing complimentary information, neuroimaging studies have been essential for understanding the impact of the AD pathological cascade on the brain. Magnetic Resonance Imaging (MRI) and Positron Emission Tomography (PET) have been integral in generating the current model of disease progression and determining the relevance of associated biomarkers (Jack and Holtzman, 2013). Although at a lower resolution than the neuropathologic examination, structural neuroimaging enables visualization of the entire brain, providing highly quantitative volumetric assessments of structural changes. Standard brain models, such as the Montreal Neurological Institute 152 non-linear 6th Generation Stereotaxic Registration Model, provide a population-based template for brain anatomy (MNI space template) that is widely used in neuroimaging for registering information across participants into a common coordinate system (MNI space) (Grabner et al., 2006; Evans et al., 2012). Functional neuroimaging studies have identified functional alterations *in vivo*, quantifying correlated activity in networked cortical regions of the brain, or “functional connectivity,” and how that connectivity changes with disease (Ibrahim et al., 2021). These analyses have revealed how specific networks may be selectively vulnerable to specific neurodegenerative processes, and demonstrated that diseases such as AD can selectively impair systems of cortico-cortical connectivity (Raichle et al., 2001; Buckner et al., 2009; Seeley et al., 2012; Dai et al., 2015; Jones et al., 2016). Such neuroimaging studies have driven an increasing appreciation for the heterogeneity of AD in the patterns of cortical and subcortical involvement (Grabowski, 2004; Gorno-Tempini et al., 2011; Murray et al., 2011; Crutch et al., 2017; Ossenkoppele et al., 2020).

Despite the complementary capabilities of neuroimaging and neuropathological studies, the two are rarely integrated systematically, limiting our ability to relate changes in structure to function in the context of neurodegenerative diseases. The published studies leveraging neuroimaging tools in the neuropathologic assessment of central nervous system diseases have largely focused on the identification of lesions visible in structural imaging that cannot be seen in gross specimens. In particular studies of neurotrauma, vascular brain injury, and multiple sclerosis have successfully employed this approach (Black et al., 2009; Bigler and Maxwell, 2011; Keene et al., 2018; Filippi et al., 2019). There have also been attempts to correlate the neuropathologic features of AD to structural brain changes identified on MRI. In general, these studies correlate broad structural changes (i.e., regional volumetrics) with overall neuropathologic assessments of AD pathology (i.e., Braak stage for neurofibrillary tangle distribution) and the methods and results have been well described in a recent review (Dallaire-Théroux et al., 2017). Nevertheless, the literature overall is lacking in systematic approaches that leverage neuroimaging tools for the precise and accurate sampling of structurally and/or functionally distinct brain regions.

In the context of brain sampling, *precision* refers to consistently targeting the same region, while *accuracy* refers to correctly targeting the intended region. Precision and accuracy in brain tissue sampling across subjects is critical to ensure the quality of brain biorepository resources for research. Standard baseline protocols for sampling brain tissue based on common landmarks are deployed across neuropathology cores of U.S. Alzheimer’s Disease Research Centers and other brain tissue banks. It is critical to implement procedures that maximize the neuroanatomical precision and accuracy of brain tissue sampling at autopsy, but it is currently unknown whether the current sampling approaches are sufficiently precise and accurate for the rapidly advancing downstream molecular technologies that utilize this tissue. Understanding limitations in this arena is essential for future progress.

Here, we establish quantitative measures of precision and accuracy of current best practice neuropathologic sampling strategies by implementing a virtual AD neuropathologic sampling protocol. Using traditional anatomic landmarks aligned with the latest NIA-AA guidelines for assessing Alzheimer’s disease neuropathologic change (Hyman et al., 2012; Montine et al., 2012), we identified four standard cortical regions of interest (ROIs) on anatomical magnetic resonance images (MRIs). We registered the results to a common stereotaxic brain space and assessed the degree of overlap between subjects (*precision*). We also sampled the same brain regions using a neuroimaging-guided approach. We compared the location of these subject-specific samples with the intended targets (*accuracy*) to demonstrate how a neuroimaging-guided approach could improve accuracy. Finally, we evaluated the functional connectivity patterns associated with the intended target and the subject-specific samples to assess the functional impact of the observed discrepancies.

## Materials and Methods

### Overview

**FIGURE 1:**
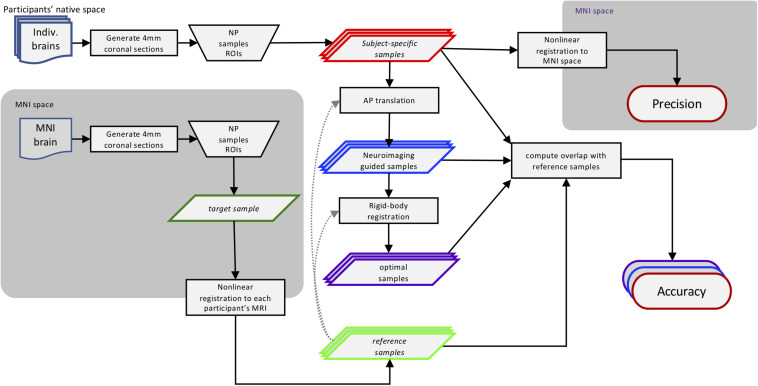
Methods workflow diagram. This diagram illustrates how the different sampling strategies were applied and then compared. Using traditional neuropathologic sampling approaches, the neuropathologist (NP) selected Regions of Interest (ROIs) in both subjects’ native MRI space (*subject-specific* samples) and standard MNI space (*target* samples). The *target* samples were then aligned to each subject’s native MRI space to form the *reference* samples. Translating the *subject-specific* sample to achieve maximal overlap with the *reference* samples resulted in the neuroimaging-guided samples and using rigid-body registration to find the highest overlap regardless of slice created the *optimal* samples. The overlap of the *subject-specific* samples from all subjects aligned in MNI space was used to evaluate the precision of traditional neuropathologic sampling. The overlap of all three sampling strategies methods with the *reference* samples was used to evaluate and compare sampling accuracy. Not shown: the functional connectivity patterns of the original *subject-specific* and *references* samples were compared to assess the potential impact of sampling variability. MRI, magnetic resonance imaging; MNI, Montreal Neurological Institute.

We virtually sampled ROIs on MR images, simulating human brain autopsies *in silico*. This approach is diagramed in Figure 1 and described in further detail below. Briefly, using the MRI scans we generated digital coronal slices of 4 mm thickness from anterior to posterior, analogous to the brain-cutting protocol of the University of Washington (UW) Biorepository and Integrated Neuropathology (BRaIN) lab and Precision Neuropathology Core. A neuropathologist (CSL) identified the regions of the brain that would routinely be sampled for neuropathologic assessment by placing appropriately sized masks (see section “Virtual Slicing and Sampling”) on the coronal MR images. The ROIs included middle frontal gyrus (MFG), middle and superior temporal gyri (MSTG), inferior parietal lobule (IPL), and primary visual (calcarine) cortex (V1). Masks placed on each subject’s native space MRI scan, i.e., *subject-specific* samples, emulate traditional neuropathologic sampling. Precision (consistently targeting the same region) was measured by registering each *subject-specific* sample to the MNI space template and computing the overlap among all such MNI-registered samples. Since the MNI space template represents stereotypical brain anatomy, a mask on this standard brain represents a *target* sample, the portion of brain tissues intended to be sampled across subjects. To determine the location of these intended regions in each subject, the four *target* samples from the MNI space template were then registered to each subject’s brain MRI, generating the *reference* samples. Accuracy (correctly targeting the intended region) was measured by comparing overlap between the *reference* and *subject-specific* samples in each subject. We also estimated potential improvements in accuracy that could be obtained with *neuroimaging-guided* samples. To do so, we translated *subject-specific* samples across each slice to maximize overlap with the reference region. This simulated process corresponds to a hypothetical, but realistically achievable, process of generating *reference* samples on MRI images and using them to guide sampling on actual tissue slices in the postmortem sampling process. Finally, to assess the potential implications of inaccuracy, we compared functional connectivity patterns of the *subject-specific* and *reference* samples due to the success of the method in delineating functional brain regions as demonstrated by high-resolution imaging studies (Glasser et al., 2016).

### Subjects

All subjects, or their designated power of attorney or legal next of kin as appropriate, consented to research, including neuroimaging studies, through protocols approved by the University of Washington (UW) Institutional Review Board. The study included 35 subjects from the UW Alzheimer’s Disease Research Center (ADRC) who underwent an MRI scan. This cohort included 14 men and 21 women, with a mean age of approximately 70 years, ranging from 54 to 91 years. The cognitive status of each individual was assessed according to clinical research criteria in the Clinical Core of the UW ADRC through structured interviews and standardized psychometric assessments. It was then determined at consensus whether the individual met criteria for cognitively normal (*n* = 21), mild cognitive impairment (*n* = 6), or dementia (*n* = 8).

### MRI Acquisition

As part of a more comprehensive imaging protocol, subjects underwent a structural MRI using a Philips 3.0T X-Series Achieva MR System (Philips Medical Systems, software version 5.1.7) with a 32-channel SENSE head coil. A structural T1-weighted 3D MPRAGE (237 axial slices, matrix size = 324 × 325, turbo-field echo factor = 181, repetition time = 10.0 ms, echo time = 4.6 ms, flip angle = 8°, shot interval = 2,709 ms) with 0.8 mm isotropic voxels was obtained for registration.

For resting state functional connectivity data, three echoes of whole-brain axial echo-planar images were collected parallel to the anterior commissure—posterior commissure (AC-PC) line (37 sequential ascending slices, 3.5 mm isotropic voxels, field of view = 224 × 224 × 129.5, repetition time = 2,500 ms, echo time = 9.5 ms, 27.5 and 45.5 ms, flip angle = 79, SENSE acceleration factor = 2.5, EPI factor = 25, 240 volumes). Each resting state scan was 240 volumes (10 min).

### MRI Analysis

Non-brain tissue was removed using FSL’s brain extraction tool (BET) (Smith et al., 2002). Multi-echo functional magnetic resonance imaging (fMRI) data were processed with an Analysis of Functional NeuroImages (AFNI) module that implements multi-echo independent component analysis (ME-ICA) to distinguish blood oxygen level-dependent (BOLD) neuronal and non-BOLD artifact components based on the characteristic linear echo-time dependence of BOLD T2^∗^ signals (Kundu et al., 2012, 2013). We registered the functional image to the structural image using boundary-based registration based on a white matter segmentation of the structural image using epi_reg in FSL (Avants et al., 2007, 2008). The structural image was registered to a population-specific template derived from an earlier pilot data set of elderly subjects within the UW ADRC clinical core, and the population-specific template was registered to the MNI space template, both using symmetric diffeomorphic registration algorithm implemented in Advanced Normalization Tools (ANTs) (Avants et al., 2008). All MRI images and registrations passed visual quality assurance checks.

### Virtual Slicing and Sampling

**FIGURE 2:**
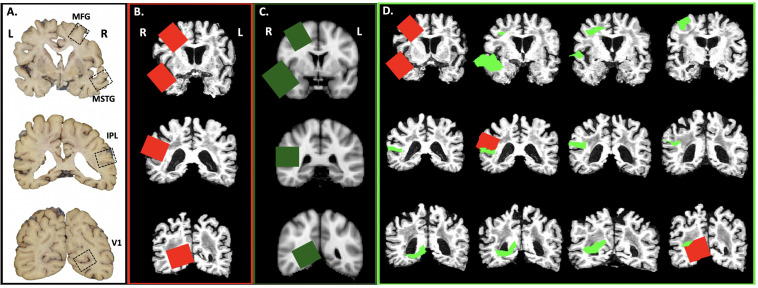
Virtual Sampling protocol. **(A)** Here we show the cortical samples routinely taken at autopsy on coronal sections of fixed human brain that are oriented by anatomic convention (MFG, middle frontal gyrus; MSTG, middle and superior temporal gyri; IPL, inferior parietal lobule; V1, primary visual cortex. For traditional neuropathologic sampling, the neuropathologist targets the regions of interest (ROIs; open black boxes) using the gyral pattern and deep brain structures as landmarks on the sliced human brain. **(B)** To mimic traditional neuropathologic sampling *in silico*, we generated coronal slices using each subject’s native space T1-weighted MRI. An example subject is shown in radiological convention. The neuropathologist identified the ROIs on the MRI slices (*subject-specific* samples; red) using the same gyral patterns and deep brain structures as would be used in physical brain slices. **(C)** The MNI space template brain was then used to create similar coronal slices and ROIs were identified as before (*target* samples, green boxes). **(D)** Finally, the ROIs identified on the coronal sections of the MNI space template brain were aligned to each subject’s T1-weighted MRI (*reference* samples, light green). A series of adjacent 4 mm thick coronal sections from a single subject’s native space T1-weighted MRI illustrates the variability between the subject-specific and *reference* samples.

Using in-house scripts written in R (R Core Team, 2017), we created images of brain slices from the structural MRI scan for each subject and the MNI template every 4 mm, corresponding to the UW neuropathology brain slicing protocol (Figure 2A). A neuropathologist (CSL) then visually inspected the slices, placing a 3.8 cm by 3.2 cm square on appropriate slices using the image editing utility GIMP^1^ to simulate sampling of the MFG, MSTG, IPL, and V1 using the traditional neuropathologic sampling approach. Once drawn, we reassembled each rectangle into a 3D mask (3.8 cm × 3.2 cm × 4 mm) to create the original *subject-specific* samples (Figure 2B).

Using the registration methods described above, the four *target* samples from the MNI space template (Figure 2C) were then registered into each subject’s native MRI space to form the *reference* samples (Figure 2D). Thus, for each ROI (MFG, MSTG, IPL, and V1) every subject had both a *subject-specific* sample, drawn on their own brain MRI in its native space according to the traditional neuropathologic sampling protocol, and a *reference* sample, obtained by transforming a reference ROI from the MNI space template sample to subject-specific space.

Anatomical regions such as gyri often extend through multiple coronal slices. While neuropathologists are unlikely to sample the wrong gyrus on a slice, not choosing the optimum slice to sample a particular portion of the gyrus across subjects likely introduces significant variability. To assess this, we next generated *neuroimaging-guided* samples by translating the *subject-specific* samples across each slice (moving in the anterior/posterior dimension) to identify the location that had maximal voxel overlap with the corresponding *reference* samples.

Another potential source of variation in sampling comes from the precise position and angle of the brain when it is prepared for slicing. Relatively slight shifts could lead to significant reductions in the amount of a *reference* sample present in any given coronal slice. To quantify the influence of how the brain is coronally sliced, we next determined the maximal voxel overlap with each *reference* sample possible for any coronal slicing. This *optimal* sample was accomplished by registering each *subject-specific* sample to the corresponding *reference* sample using rigid body registration (Jenkinson and Smith, 2001; Jenkinson et al., 2002), which allows translation and rotation, but not scaling, shearing, or warping.

**FIGURE 3:**
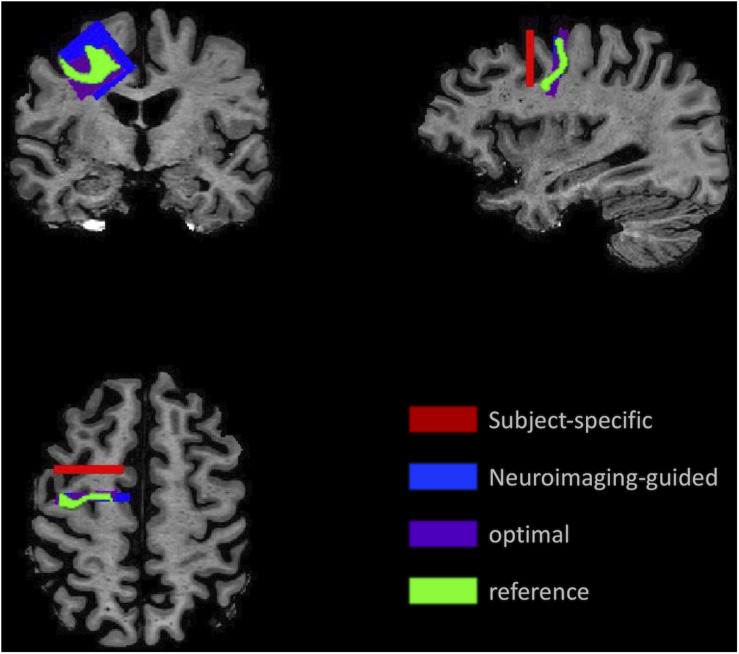
Illustration of the sampling framework supporting assessment of precision and accuracy. Position of the samples demonstrates the variation in location and angle of the MFG ROI for the *subject-specific* samples (red) generated using traditional neuropathologic sampling and the *reference* sample (light green) which indicated the intended target. Using the *reference* sample to guide slice sampling results in the *neuroimaging-guided* sample (blue), which shows marked improvement in overlap with the *reference* sample. The highest overlap that could be achieved with slicing at any angle or coronal position is the *optimal* sample (purple).

An example of each type of sample for the MFG in a single subject’s MRI is illustrated in Figure 3, which highlights the variability in the samples.

### Functional Connectivity

For each of the four ROIs, seed-based functional connectivity from the *subject-specific* samples and the *reference* samples were calculated for 264 brain regions that were previously identified as functionally unique (Power et al., 2011). We calculated the Pearson correlation of the average timeseries in each sample to the average timeseries of each of the 264 brain regions. This produced two sets of whole-brain functional correlations, one for the *subject-specific* samples and one for the *reference* samples. For each of the four ROIs, we assessed whether there was a statistically significant difference in the number of subjects showing increased or decreased connectivity with each of the 264 brain regions (see section “Functional Connectivity”). All scripts and workflow were implemented reproducibly using GNU Make (Askren et al., 2016).

### Statistical Analysis

#### Anatomic Overlap

To assess sampling precision (degree of overlap between subjects), we calculated the number of people whose *subject-specific* masks overlapped in MNI space at each voxel. This descriptive metric illustrates the degree to which the same anatomical region is sampled across different individuals.

For each sampling method (traditional neuropathologic, neuroimaging-guided, and optimal), we assessed accuracy by comparing the percent overlap of each ROI with the corresponding *reference* sample using Poisson regression with robust standard errors. For each ROI, the percent overlap with the *reference* sample found for the *subject-specific* samples was compared to the percent found for the *neuroimage-guided* samples, and the percent with the *neuroimaging-guided* samples was compared to *optimal* samples, using the Wilcoxon matched-pairs signed-rank test.

#### Functional Connectivity

To examine whether functional connectivity patterns significantly differed between each of the four corresponding *subject-specific* and *reference* samples, we used non-parametric permutation testing (with 1,000 samples) to create a distribution of expected differences in functional connectivity between the sample types. We then calculated the empirical threshold for the top and bottom 5% differences for each reference region.

## Results

### Precision: Variation in Sampling Across Individuals for All Four Regions of Interest

**FIGURE 4:**
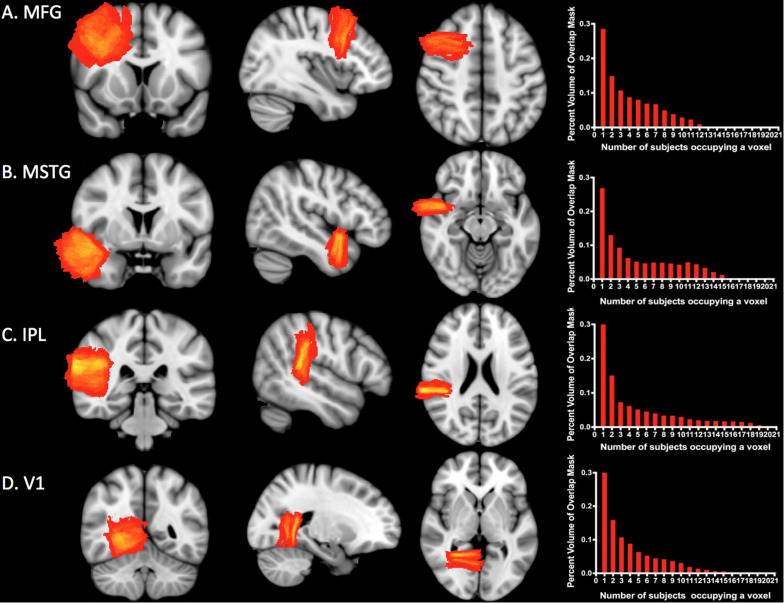
Sampling precision. Heat maps and histograms conveying of the variation in sampling for each ROI. Each heat map shows the degree of overlap for the ROI. The red end of the spectrum represents less overlap and the yellow end represents greater overlap. The histograms illustrate the number of subjects whose simulated neuropathological blocks overlap in the MNI template space and the percentage of voxels with each degree of overlap. The maximum amount of overlap for any given voxel was only 12 of 35 subjects for MFG **(A)**, 15 of 35 for MSTG **(B)**, 21 of 35 for IPL **(C)**, and 15 of 35 for V1 **(D)**. Approximately 30% of each ROI mask was devoid of any overlap. MFG, middle frontal gyrus; MSTG, middle and superior temporal gyri; IPL, inferior parietal lobule; V1, primary visual cortex.

Figure 4 shows the overlap for *subject-specific* samples for each ROI across individuals in the MNI-space template. At least two subjects overlapped per voxel in 71% of MFG, 73% of MSTG, 67% of IPL, and 68% of V1. The maximum number of subjects who overlapped in any given voxel was only 12 of 35 subjects for MFG, 15 of 35 for MSTG, 21 of 35 for IPL, and 15 of 35 for V1, though in each case this overlap comprised less than one percent of the total mask volume (see Figure 4, right column).

### Accuracy: Localization Discrepancy of Each ROI Between Traditional Neuropathologic Sampling and Neuroimaging-Guided Sampling

**FIGURE 5:**
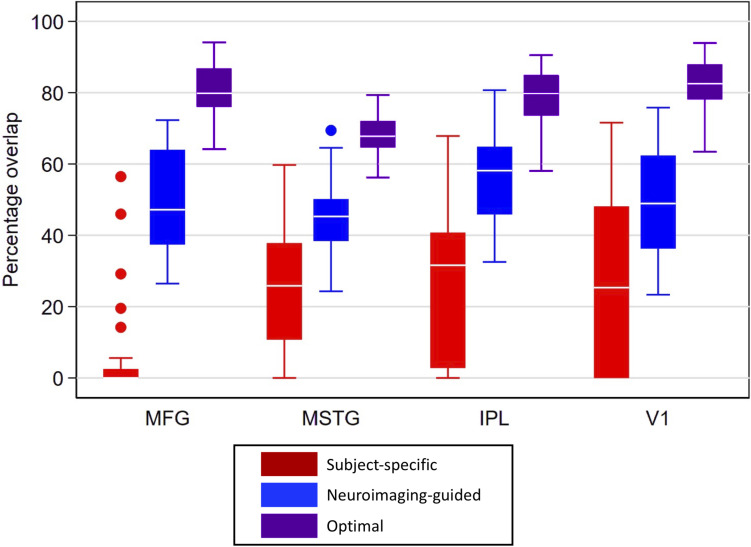
Box plots for percentage overlap by method and region. Each box spans the 25th to 75th percentiles, with the mean indicated. The whiskers define 1.5 times the inter-quartile range; individual observations more extreme than this are indicated with dots. Image-guided sample selection, including both adjusting the anterior-posterior location of the selection as well as optimizing the angle markedly improved overlap with the reference sample compared to the overlap of the standard sample. MFG improved from 5.2% in the *subject-specific* sample to 80% in the *optimal* sample, MSTG improved from 24 to 68%, IPL improved from 27 to 80%, and V1 improved from 26.5 to 83%. MFG, middle frontal gyrus; MSTG, middle and superior temporal gyri; IPL, inferior parietal lobule; V1, primary visual cortex.

Overlap statistics showed that *subject-specific* samples from the traditional neuropathologic sampling approach had limited overlap with the *reference* samples regardless of the brain region being sampled [means (standard deviation) of 5.2% (13.1%) for MFG, 24.0% (16.0%) for MSTG, 27.0% (21.1%) for IPL, and 26.5% (25.0%) for V1; see Figure 5]. There was significantly lower overlap in MFG than all other regions (*p* < 0.001), which appears to be largely due to variation of the *subject-specific* samples in the anterior-posterior dimension. The *neuroimaging-guided* sampling approach more accurately localized to the *reference* samples, significantly improving the overlap for each ROI [means (standard deviation) of 50.0% (15.4%) for MFG, 45.7% (10.1%) for MSTG, 55.7% (12.7%) for IPL and 48.4% (15.5%) for V1; Figure 5 shows the distribution]. Optimizing the position and angle of the coronal brain sections accounts for approximately 20–30% of the remaining difference between the target and sampled regions, with ROIs for the *optimal* neuroimaging-guided sampling approach ultimately achieving mean overlaps ranging from 68 to 83% (Figure 5). In each region, the neuroimaging-guided sampling method significantly outperformed the traditional neuropathology sampling approach and optimizing the coronal slicing further improved accuracy (*p* < 0.0001 for each comparison).

### Functionally Relevant Consequences of ROI Sampling Discrepancies Are Most Pronounced in the Middle Frontal Gyrus

**FIGURE 6:**
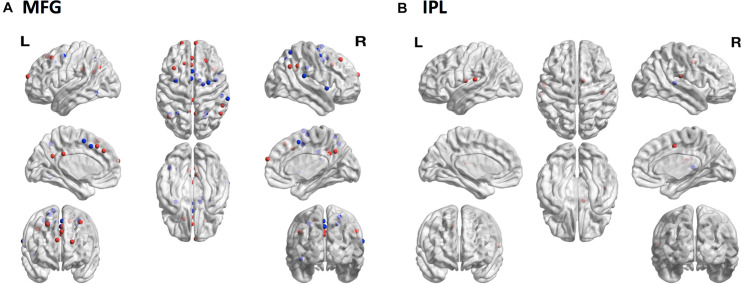
Consequences of sampling variability. The locations of 264 brain regions showing differences in seed-based functional connectivity patterns from the *subject-specific* samples relative to the *reference* samples. **(A)** In MFG there were 15 regions with decreased (blue dots) and 15 regions with increased (red dots) connectivity to the *subject-specific* sample compared to the reference sample. **(B)** In IPL one region showed decreased (blue dot) and four regions showed increased (red dots) connectivity to the *subject-specific* sample compared to the reference sample. MFG, middle frontal gyrus; IPL, inferior parietal lobule.

To assess whether sampling variability could result in selecting regions functionally distinct from the target region, we compared patterns of functional connectivity from the *subject-specific* samples to patterns of functional connectivity from the *reference* samples for each of the four ROIs. The MFG showed the greatest discrepancy with 15 regions showing decreased connectivity and 15 regions showing increased connectivity from the *subject-specific* sample compared with the *reference* sample (Figure 6A). There were also differences among the IPL samples, with one region showing decreased connectivity and four regions showing increased connectivity (Figure 6B). Neither MSTG nor V1 showed any significant differences in functional connectivity between the *subject-specific* and *reference* samples.

## Discussion

Using an MRI-based simulation, this study estimates the variability of traditional neuropathologic tissue sampling and serves as a benchmark against which other sampling protocols can be compared. It also highlights how neuroimaging tools could be leveraged to increase the consistency of neuropathologic sampling and improve the targeting of specific brain regions by mapping neuroimaging-identified ROIs onto slices of postmortem human brain.

In our virtual sampling protocol, we found limited overlap across individuals for the majority of sampled ROIs when using traditional neuropathologic sampling strategies. This lack of overlap was largely due to variation in the anterior-posterior (AP) localization when selecting the target region. For example, the MFG is normally sampled from the coronal section that also has the most complete view of the anterior commissure, but when MFG was selected on the MNI space template using this approach, and then mapped to each individual MRI, there was significant variability in the AP location of this brain region relative to the location of the anterior commissure. Indeed, when registered to each subject’s native space MRI scan, the generated *reference* sample rarely mapped to the same coronal slice as the anterior commissure, which is where the *subject-specific* sample was identified by the traditional neuropathologic sampling approach.

While the MFG had the most pronounced variability, all ROIs showed low degrees of overlap that could be significantly improved by adjusting the slice from which the sample was taken. Using the *reference* sample as a guide to select a slice to sample in each subject’s native MRI space increased overlap with the *reference* sample by 20–45 percent, sometimes resulting in a location 2 cm from the originally selected sample. These results suggest that relying on gross neuropathological landmarks alone to select samples at autopsy leads to significant variability, even when sampled by the same neuropathologist. Further, this exercise highlights how sampling precision could be enhanced if neuroimaging were integrated with the standard neuropathologic assessment.

Although neuroimaging-guided ROI selection improved the localization of the intended target, it did not completely resolve the discrepancy with the *reference* samples. An additional 20–30% improvement was achieved by optimizing (sub-slice) sample position and the angle of rotation. There was no apparent pattern to the angle adjustment needed to improve overlap, but better methods for standardizing the coronal axis prior to coronally slicing the brain could reduce this source of variability. The remaining discrepancy (∼20%) is primarily due to individual non-linear anatomical differences, though small errors in registration cannot be ruled out. Although it is beyond the scope of this exercise to fully investigate, some of the individual differences seen here may in part be related to underlying pathology. This sample is representative of individuals who donate tissue to an Alzheimer’s Disease Research Center and some of the participants had mild cognitive impairment or dementia while the majority had normal cognition. Neither AD biomarkers nor definitive post-mortem neuropathologic data was available to confirm the association between these clinical diagnoses and the underlying pathology. However, these will be important questions to pursue in subsequent work to further extend the utility of integrated neuroimaging and neuropathological techniques.

Our model suggests that the discrepancies in ROI selection have meaningful consequences for targeted sampling of functionally distinct regions, demonstrated by comparing the functional connectivity patterns of *subject-specific* and *reference* regions for each ROI. Some brain structures, such as the MFG, extend multiple centimeters in the AP dimension and contain heterogeneous brain areas (Glasser et al., 2016). As a result, the cell-types, connectivity patterns, and characteristic pathology that define the sampled region may vary significantly depending on the coronal level at which it is sampled. The spatial differences between the *reference* and *subject-specific* samples ranged up to 20 mm, and the differences in functional connectivity we observed suggest that the traditional neuropathologic sampling approach sometimes leads to sampling functionally distinct brain tissue across individuals. The MFG showed the greatest discrepancy in connectivity profiles, consistent with the observation that it had the least amount of physical overlap across sampled brains compared to the other ROIs evaluated in this study. The IPL also showed discrepant connectivity patterns between the two samples. This is in contrast to MSTG and V1, regions with similar overlap discrepancies between the *subject-specific* and *reference* samples as the IPL, but for which we did not demonstrate differences in functional connectivity. This may be explained by observed differences in the cortical parcellation of these different anatomical regions. Data from multi-modal cortical parcellation studies of the Human Connectome Project show numerous subdivisions in MFG and IPL brain regions from anterior to posterior (Glasser et al., 2016). Therefore, even relatively small physical sampling discrepancies could lead to effectively sampling functionally distinct regions of the cortex. Conversely these parcellations show larger AP homogeneity for V1 and MSTG, suggesting that the observed patterns of variation in sampling would be less likely to result in differences in connectivity patterns. Potential implications for this sampling variability include the negative effects on downstream research applications, which are moving increasingly toward single-cell profiling of specific brain regions (Lein et al., 2017; Regev et al., 2017; Hodge et al., 2019). Generating useful data from single cell-omics profiles across different individuals will depend in part on the ability to reliably obtain samples from specific functional brain regions.

Our study had several limitations, one of which concerns the subjects within the cohort, which includes a range of older adults, some of whom have cognitive impairment or AD dementia and associated cortical atrophy. Although we minimized registration error by using a population-specific template, individual differences, and residual registration variability may have contributed to some discrepancies between the *subject-specific* and *reference* samples. We used ANTs to register subjects’ structural images to a standard template, treating these registration results as a gold standard; however, with even the most advanced registration algorithms, alignment of structural features can differ by 2–5 mm (Askren et al., 2016). Functional variability may contribute to a second level of variability in this study, since neuropathological burden affects functional connectivity and is heterogeneous in elderly adults (Sonnen et al., 2009; Dowling et al., 2011). This could diminish the statistical power of our ability to detect differences in functional connectivity, thus, our results may be an underestimate of the true consequences of sampling variability. Even when anatomical landmarks are perfectly aligned, variability in the borders of functionally distinct cortical areas can be up to 10 mm (Eickhoff et al., 2009).

Finally, our results represent a best-case scenario for tissue sampling and future work will need to extend this to real-word scenarios. First, all samples were selected by the same board-certified neuropathologist, and although not specifically analyzed in this model, sampling variability between neuropathologists/technicians would likely contribute additional sampling variability. Second, all “sampling” was performed *in silico* and not using actual postmortem human brain tissue. Because MRI scans, unlike brain slices, have no *ex vivo* or fixation distortion, further assessment of the actual sampling of physical brain slices must be done to address potential confounding effects related to *ex vivo* and fixation-related tissue distortions in registering images of brain slices to the MRI. It may be that *ex vivo* postmortem imaging is necessary to provide a bridge between the antemortem neuroimaging studies and the postmortem tissue sampling.

This study represents an initial attempt to use neuroimaging tools to estimate the precision accuracy of current brain tissue sampling practices and promote more accurate targeting of specific cortical regions. This is an important step in establishing robust links between neuropathologic and neuroimaging-derived measures. Innovative approaches to the study of AD are being developed in both neuropathology and neuroimaging; an interdisciplinary strategy leveraging the strengths of both approaches is more likely to foster success in advancing our understanding of AD. We envision a model whereby studies conducted in postmortem human brain tissue are informed by antemortem assessments, with tools such as fMRI leveraged to localize functional regions and postmortem MRI serving as a bridge connecting brain imaging to tissue sampling (Tregidgo et al., 2020). Integration could be achieved through registration across all modalities to a common neuroanatomical reference system, such as MNI space or a common coordinate framework. For example, future extensions of the methods described here could include assessing neuropathologic sampling relative to high-resolution multimodal MRI parcellations (Glasser et al., 2016) to provide more detailed insight into the impact of various aspects of sampling variability. We expect that incorporating such integrative approaches into prospective cohort studies will enhance research rigor and reproducibility, increase the probability for novel insights, contribute to mechanistic hypotheses for testing in model systems, and foster innovative diagnostic and therapeutic strategies.

## Data Availability Statement

The raw data supporting the conclusions of this article will be made available by the authors, without undue reservation.

## Ethics Statement

The studies involving human participants were reviewed and approved by the University of Washington Institutional Review Board. The patients/participants provided their written informed consent to participate in this study.

## Author Contributions

CL, TM, CK, and TG contributed to the development of the project. CL and TM performed the initial data collection and analysis. LG performed additional statistical analyses. CL, JW, and TM contributed to the figure production. All authors contributed significantly to writing and editing.

## Conflict of Interest

The authors declare that the research was conducted in the absence of any commercial or financial relationships that could be construed as a potential conflict of interest.

## Publisher’s Note

All claims expressed in this article are solely those of the authors and do not necessarily represent those of their affiliated organizations, or those of the publisher, the editors and the reviewers. Any product that may be evaluated in this article, or claim that may be made by its manufacturer, is not guaranteed or endorsed by the publisher.

## Footnotes

1
https://www.gimp.org/
